# Secular trends in testosterone- findings from a large state-mandate care provider

**DOI:** 10.1186/s12958-020-00575-2

**Published:** 2020-03-09

**Authors:** Gabriel Chodick, Shdema Epstein, Varda Shalev

**Affiliations:** 1 Sackler Faculty of Medicine, Tel Aviv University, Tel Aviv, Israel; 2 Maccabi Institute for Research & Innovation, Maccabi Healthcare Services, HaMered 27, 68125 Tel Aviv, Israel

**Keywords:** Testosterone, Secular trends, Israel

## Abstract

**Background:**

Several studies from the US and Europe have shown a population-level decline in serum testosterone in men from 1970’s to early 2000’s. However, to the best of our knowledge, no study examining population-level decline in testosterone has been published in more recent years. The study objective was therefore to examine secular trends in testosterone levels among Israeli men in the first and second decades of the twenty-first century,

**Methods:**

All incident total testosterone performed between1/2006 and 3/2019 among 102,334 male members of a large health organization.

**Results:**

A significant (*p* < 0.001) and prominent trend of age-independent decline in the testosterone levels was recorded during the study period for most age groups.

**Conclusions:**

There was a highly significant age-independent decline in total testosterone in the first and second decades of the twenty-first century. The decline was unlikely to be explained by increasing rates of obesity.

## Background

Testosterone plays a major role in male reproductive function, including stimulating Sertoli cell function and spermatogenesis, as well as affecting non-reproductive organs such as muscle growth, stimulating bone mineralization, erythropoiesis, and cognitive function [1–3].

Several studies from the US [4, 5] and Nordic countries [6, 7] have shown a significant decline in serum testosterone among men from 1970s to early 2000s. However, to the best of our knowledge, no such data were published after 2004. Changes in lifestyle and health indices have been associated with declining testosterone, free testosterone, and SHBG levels, including body weight gain [5] and smoking cessation [5, 8]. Since the decline in testosterone levels seem to be affected by modifiable risk factors, the objectives of the current study were to assess long term trends over recent decades in total testosterone lab results using data from a stable, population-based cohort of male members of a large state-mandated health provider in Israel.

## Methods

This cross sectional study was conducted in Maccabi Healthcare Services (MHS), the second largest health organization maintenance in Israel, serving 25% of the total population countrywide (2.3 million members). According to the 1994 Israel National Health Act, MHS may not bar applicants on any grounds, including age or state of health. Thus, all sectors of the Israeli population are represented in MHS, except for young adults aged 18–21, since a high percentage of them are enlisted in the Israeli Defense Forces (IDF), and receive medical care there. Membership retention rate in MHS is very high (less than 1% is leaving the organization annually) allowing for a long retrospective follow-up with a minimal lost to follow-up.

### Testosterone tests

We pulled data on all the blood test measures of total testosterone (*Current Procedural Terminology*, 4th Edition code 84002) performed on men aged 13–80 between the years 1/2006–3/2019. Included in the analysis were only the first blood sample taken from each patient ever since 2000. We additionally filtered the data such that all included samples have the same lab norms (8.4–28.7 nanomole/liter), to ensure that all the samples were measured using the same lab methods (all the blood tests in Maccabi are analyzed at a single central lab).

### Statistical methods

The study protocol has been approved by the Maccabi Healthcare Service’s institutional review board. Differences in age-specific mean BMI between decades were compared using η^2^, a measure of effect size using analysis of variance (ANOVA). To test the significance of the year as a contributing factor to the testosterone level beyond age we fit a quadratic model for the age and a linear model for the measurement year (*testosterone level* = 1 + *age* + *age*^2^ + *age*^3^ + *age*^4^ + *year*, the quadratic model was used for age due to the strictly non-linear behavior of testosterone as a function of age in the range 13–80). All analyses were conducted using IBM-SPSS ver. 25, MATLAB, and R (R: A language and environment for statistical computing. R Foundation for Statistical Computing, Vienna, Austria. 2018).

## Results

**Fig. 1:**
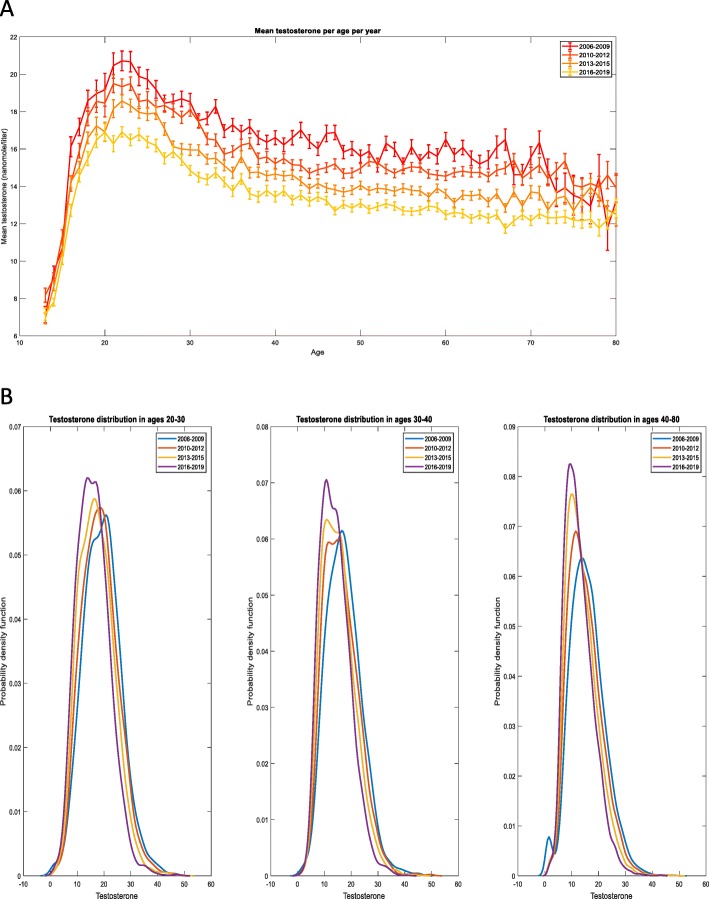
Trend of testosterone serum levels per age between 2006 and 2019. (**a**) Mean total testosterone in serum (nanomole/liter) for patients in a given age for the years 2006–2019 grouped to four year-groups, error bars show standard deviation of the mean. (**b**) Serum total testosterone values (nanomole/liter) distributions within three age groups over the years

The final analysis was performed on a total of 102,334 eligible patients (mean age 45.6, SD = 17.3, Table 1). No meaningful differences in BMI (η^2^ = 0.001) or in exact age (η^2^ = 0.004) between study periods were observed. Age-specific testosterone levels over the observation period are depicted in Fig. 1a. At age 21, at testosterone peak level, levels declined from 19.68 in 2006–9 to 17.76 in 2016–19. Fig. 1b presents the full distribution of age-specific testosterone values by time period (estimated using kernel density estimation). Both show a prominent trend of age-independent decline in the testosterone levels for most age groups. The results were highly significant (*p* − *value* < 0.001). In addition, to account for the imperfect polynomial fit we fit a linear model (*testosterone level* = 1 + *age* + *year*) separately for the ages range 13–22,23–35 and 36–80 where the behavior of the testosterone level as a function of age is approximately linear, with similarly significant results (*p* − *value* < 0.001).

**Table 1:** Mean BMI and age of study population by period

Period												
Age group		2006–9			2010–2			2013–5			2016–9	
	n	Mean	SD	n	Mean	SD	n	Mean	SD	n	Mean	SD
Age (y)												
13–18	1979	14.66	1.35	1900	14.76	1.344	2374	14.77	1.336	2890	14.71	1.336
18–24	1163	21.40	2.11	1549	21.56	2.079	1992	21.38	2.107	2570	21.52	1.993
25–34	3400	30.10	2.82	3749	29.8	2.828	4194	29.77	2.819	5448	29.56	2.859
35–44	3643	39.29	2.86	4900	39.67	2.835	4999	39.88	2.848	5568	39.83	2.919
45–54	3795	49.61	2.83	5815	49.66	2.859	6018	49.56	2.837	7917	49.57	2.832
55–64	3812	59.15	2.76	6445	59.48	2.862	5882	59.45	2.883	7111	59.35	2.884
65–74	1821	68.99	2.74	3489	68.74	2.903	3744	68.47	2.671	5258	68.94	2.71
75–84	492	78.15	2.63	992	77.9	2.59	1162	78.23	2.648	1672	78.51	2.668
85+	72	87.76	2.93	129	87.52	2.447	154	87.84	3.289	267	87.49	2.8
Total	20,177	43.79	17.27	28,968	47.22	17.047	30,519	46.22	17.68	38,701	46.83	18.095
BMI (kg/m2)												
13–18	1905	21.70	5.80	1821	21.45	5.351	2165	21.44	5.498	2135	21.03	5.371
18–24	1052	24.67	5.13	1352	24.82	5.218	1452	24.54	5.268	1051	24.81	5.739
25–34	3140	26.38	4.83	3322	26.05	4.991	3064	26.11	5.098	2167	26.48	5.453
35–44	3472	27.58	4.79	4525	27.57	5.025	4050	27.49	5.062	2593	28	5.382
45–54	3725	28.07	4.55	5633	28.35	4.749	5271	28.37	4.795	4231	28.99	4.97
55–64	3753	28.40	4.37	6353	28.53	4.448	5474	28.51	4.542	4458	28.84	4.671
65–74	1785	28.01	4.18	3454	28.07	4.2	3670	28.18	4.327	4028	28.27	4.423
75–84	447	27.55	4.12	966	27.28	3.916	1123	27.41	3.798	1316	27.43	4.212
85+	47	26.30	3.88	115	26.89	4.134	138	26.1	3.463	190	25.84	3.587
Total	19,326	26.94	5.13	27,541	27.28	5.078	26,407	27.15	5.207	22,169	27.38	5.464

## Discussion

In a large population of Israeli patients referred for a testosterone blood test during the first and second decades of the twenty-first century, there was a highly significant age-independent decline in total testosterone. The decline was unlikely to be explained by increasing rates of obesity. These results are in accordance with previous studies which showed a secular decline in testosterone serum levels in earlier years (1970’s to 2000’s) in other countries (Table 2).

**Table 2:** Previous studies on secular trends in serum testosterone in men

Source	Design	N	Period	Parameters adjusted for	Is age-adjusted decline significant?	
					before adjustment	after adjustment
Perheentupa et al. 2013 [7]	Observational, Finnish National Public Health Institute	3271	1972–2002	BMI	Yes	Yes
Mazur et al. 2013 [9]	Longitudinal cohort. US Air-Force veterans	991	1982–2002	BMI	Yes	Yes
Andersson et al. 2007 [6]	Population surveys at Glostrup Uni. Hosp., Denmark	5350	1982–2001	BMI	Yes	No
Travison et al. 2007 [5]	Random sample MA, USA	1532	1987–2004	BMI, smoking, medications, chronic illnesses, employment, marital status, general health	Yes*	Yes
Nyante et al. 2012 [4]	Observational NHANES participants	2315	1988–2004	BMI\body fat, waist circumference, ethnicity, alcohol use, smoking, chronic illnesses, general health, medications	Yes	No
Walsh et al. 2015 [10]	Observational. VA HCS, Pacific NW	44,762	2002–2011	None	Yes	NA**

*Age matched trend of approx.-1.2% per year **Not calculated. Among tested patients, there was an observed monotonous increase in the proportion of patients with low T level from 35% in 2002 to 47.3% in 2011. ** An increase in SHBG remained significant. *NHANES* National Health and Nutrition Examination Survey; *VA* Veterans Affairs

In this analysis we did not adjust for BMI, as this is unclear whether BMI is a potential mediator or confounder since BMI has not been established as a sole explaining parameter in previous studies on longitudinal trends of testosterone. Analysis performed on the research population with available data on BMI showed little variation (< 1 kg/m^2^) in the mean age-specific BMI between study periods, with no discernible trend (data not shown). We therefore suggest that the observed testosterone decline is not likely to be explained by obesity trends.

Other comorbid conditions which have be associated with low serum testosterone, include heart failure, osteoporosis, dyslipidemia, and type 2 diabetes [11]. While the prevalence of these chronic diseases has been increasing over recent decades, it is unlikely to explain increased rate of hypogonadism across all age groups as indicated by our results. Vitamin D deficiency has also been linked to low total testosterone; In light of data demonstrating a marked decrease in serum 25(OH) D levels in the US [12], this potentially important etiology should be further explored.

In two of the previous observational reports [2, 4] adjustment to body mass index (BMI) led to a nullification of the period-related changes in testosterone. However, in the other two observational reports [1, 3] the age-specific testosterone decline remained significant after adjustment to BMI. Additionally, an US longitudinal study [13] of 991 men have shown that a between the years 1982–2002 testosterone decreased more than expected by aging. Decline was evident also in men who did not gain weight during the study. Thus, it cannot be concluded based on previous studies that the secular testosterone decline can be explained by a concurrent secular increase in body weight.

All the patients observed in this study were referred to a testosterone blood test by a physician while the indication for the referral was not available in study. While this is an obvious limitation of the study, particularly of its external validity, there is no reason to believe it affect internal validity, as the indications for the test have not been changed through the observation period. However, there still may be a concern that the observed trend can be explained by a growth in the size of the subpopulation of referred patients that end up having a below-norm level of testosterone in the serum, i.e. that the trend is due to a growth in the number of patients with a discernible problem rather than a decline of testosterone levels in the general healthy population. In order to address this concern, we repeated the analysis only for the samples which were within the normal range with similar results (data not shown).

It should also be noted regarding the external validity that the particularly large sample size in this study leads us to believe that in spite the aforementioned limitation the results can still be reasonably generalized to the general population, especially as most patients referred to this test eventually are not diagnosed with a discernible medical problem affecting the level of testosterone in the serum. Moreover, the age-specific levels of circulating testosterone are comparable with previous reports, including a study on 58,162 consecutive results in men from a single large pathology laboratory in Australia [14].

## Conclusions

The results of this large real-world data analysis corroborate previous scattered reports that mean testosterone for men in developed countries is decreasing, which is unlikely to be explained by increasing rates of obesity. The biological mechanisms of this disquieting secular trend should be further examined.

## Data Availability

The datasets generated and/or analyzed during the current study are not publicly available due to privacy regulations but are available from the corresponding author on reasonable request.

## Abbreviations

BMI: Body mass index

## References

[1] Finkelstein JS, Lee H, Burnett-Bowie SA, Pallais JC, Yu EW, Borges LF, Jones BF, Barry CV, Wulczyn KE, Thomas BJ, Leder BZ (2013). Gonadal steroids and body composition, strength, and sexual function in men. N Engl J Med.

[2] Finkelstein JS, Lee H, Leder BZ, Burnett-Bowie SA, Goldstein DW, Hahn CW, Hirsch SC, Linker A, Perros N, Servais AB, Taylor AP, Webb ML, Youngner JM, Yu EW (2016). Gonadal steroid-dependent effects on bone turnover and bone mineral density in men. J Clin Invest.

[3] Blazer D, Liverman C. Testosterone and aging: clinical research directions. In: Blazer DG, Liverman CT, editors. Testosterone and aging: clinical research directions; Mar 12. Washington (DC): National Academies Press; 2004. 25009850

[4] Nyante SJ, Graubard BI, Li Y, McQuillan GM, Platz EA, Rohrmann S, Bradwin G, McGlynn KA (2012). Trends in sex hormone concentrations in US males: 1988-1991 to 1999-2004. Int J Androl.

[5] Travison TG, Araujo AB, Kupelian V, O'Donnell AB, McKinlay JB (2007). The relative contributions of aging, health, and lifestyle factors to serum testosterone decline in men. J Clin Endocrinol Metab.

[6] Andersson AM, Jensen TK, Juul A, Petersen JH, Jorgensen T, Skakkebaek NE (2007). Secular decline in male testosterone and sex hormone binding globulin serum levels in Danish population surveys. J Clin Endocrinol Metab.

[7] Perheentupa A, Makinen J, Laatikainen T, Vierula M, Skakkebaek NE, Andersson AM, Toppari J (2013). A cohort effect on serum testosterone levels in Finnish men. Eur J Endocrinol.

[8] Camacho EM, Huhtaniemi IT, O'Neill TW, Finn JD, Pye SR, Lee DM, Tajar A, Bartfai G, Boonen S, Casanueva FF, Forti G, Giwercman A, Han TS, Kula K, Keevil B, Lean ME, Pendleton N, Punab M, Vanderschueren D, Wu FC (2013). Age-associated changes in hypothalamic-pituitary-testicular function in middle-aged and older men are modified by weight change and lifestyle factors: longitudinal results from the European male ageing study. Eur J Endocrinol.

[9] Mazur A, Westerman R, Mueller U. Is rising obesity causing a secular (age-independent) decline in testosterone among American men? PLoS One. 2013;8(10):e76178. doi: 10.1371/journal.pone.0076178. 10.1371/journal.pone.0076178 PMC3797769 24146834

[10] Walsh TJ, Shores MM, Fox AE, Moore KP, Forsberg CW, Kinsey CE, Heckbert SR, Zeliadt S, Thompson ML, Smith NL, Matsumoto AM (2015). Recent trends in testosterone testing, low testosterone levels, and testosterone treatment among Veterans. Andrology.

[11] Livingston M, Kalansooriya A, Hartland A, Ramachandran S, Heald A (2017). Serum testosterone levels in male hypogonadism: why and when to check-a review. Int J Clin Pract.

[12] Ginde A, Liu M, Camargo CA (2009). Trends of vitamin D insufficiency in the US population, 1988-2004. Arch Intern Med.

[13] A Longo P, Kavran JM, Kim MS, Leahy DJ 2013 Generating mammalian stable cell lines by electroporation. Methods Enzymol 529:209–226. 10.1016/B978-0-12-418687-3.00017-3 PMC4088271 24011048

[14] Handelsman D, Sikaris K, Ly L (2016). Estimating age-specific trends in circulating testosterone and sex hormone-binding globulin in males and females across the lifespan. Ann Clin Biochem.

